# Analgesic efficacy of ketorolac associated with a tramadol/acetaminophen combination after third molar surgery - a randomized, triple-blind clinical trial

**DOI:** 10.4317/medoral.22744

**Published:** 2018-12-24

**Authors:** Luciana-Dorochenko Martins, Márcia Rezende, Alessandro D. Loguercio, Marcelo-Carlos Bortoluzzi, Alessandra Reis

**Affiliations:** 1 DDS, MS, PhD, Professor. School of Dentistry, State University of Ponta Grossa, Ponta Grossa, Paraná, Brazil; 2 DDS, MS, PhD, Professor School of Dentistry. Paulo Picanço School of Dentistry, Fortaleza, Ceará, Brazil

## Abstract

**Background:**

This study compared the efficacy of ketorolac alone versus its combination with tramadol/acetaminophen for pain control after mandibular third molar surgery.

**Material and Methods:**

A randomized, triple-blind clinical trial was carried out with 52 patients divided into 2 groups: Group K+T+A (1 tablet of Ketorolac 10 mg plus and 1 capsule of Tramadol 37.5 mg/acetaminophen 325 mg) and Group K (1 tablet of Ketorolac 10 mg plus and 1 placebo capsule). The treatments were given 1 h before the surgery and was repeated 4 times per day, for 48 h. The difference in postoperative pain was assessed by 4 primary end-points: pain intensity (VAS 100mm, for 48 h), rescue medication, overall assessment and adverse effects.

**Results:**

Significant differences in pain intensity were observed in the different times (*p*<0.05). The comparison of groups in each time showed significant differences only of 9 h, with lower level of pain intensity for group K+T+A (*p* = 0.005). The need of analgesics was higher in Group K (*p*<0.001), the need of antiemetic were greater in Group K+T+A (*p*<0.0001). No significant difference between groups were observed in overall assessment. The adverse effects was higher in Group K+T+A.

**Conclusions:**

The current study showed that both ketorolac and the combination of ketorolac plus tramadol/acetaminophen showed good control of pain after the extraction of the lower third molars. Although the combination group showed lower pain at 9 h, the difference is small and not clinically relevant.

** Key words:**Ketorolac, molar, third, surgery, oral, Tramadol, Acetaminophen.

## Introduction

Third molar surgery is often accompanied by postoperative complications such as pain, buccal swelling and trismus (1). Among these, pain is one of the most common and significant postoperative complications, and it mainly arise from inflammatory response (2).

Several biochemical mediators are involved in the pain process, particularly histamine, bradykinin and prostaglandins (3). The intensity of postoperative pain ranges from moderate to severe during the first 24 h after surgery, with the pain peak being within the first 12 h when a medium-acting local anesthetic is used (4).

Numerous studies have investigated alternatives for the management of pain and discomfort generated by third molar surgery (5). Several analgesics have been used for this purpose, including nonsteroidal anti-inflammatory drugs and some opioids (3).

Among the nonsteroidal anti-inflammatory drugs investigated, ketorolac is one of the pharmacological options available, it is reported to have a potent analgesic effect similar to opioids (6) as well as a moderate anti-inflammatory activity (7), which seems adequate for the treatment of moderate-to-severe acute pain (8). Due to these characteristics, this drug has been investigated for pain control after third molar surgeries.

Despite these promising findings, rescue medication for pain relief was still necessary, even with the administration of ketorolac, which suggests that preoperative administration is not enough to eliminate postoperative pain (9,10).

Combining analgesics may provide greater analgesia than the individual agents through the synergistic action of the individual drugs (11), and allowing the use of lower doses for each medication may improve the patients’ tolerability (9,11). However, among the several number of possible drug combinations, there is a lack of knowledge regarding which combination and the respective drug dosages have a better analgesic efficacy (12).

Perhaps combining a nonsteroidal anti-inflammatory drug (NSAID) such as ketorolac with opioid analgesics such as tramadol/acetaminophen may lead to lower postoperative pain. Clinical studies have reported that the combination of tramadol/acetaminophen 37.5 mg/325 mg was effective and well tolerated in patients with dental pain (13).

Third molar surgery pain is an excellent clinical model for acute pain (4). Pain of this type is predictable, generally acute and of moderate-to-severe intensity. To the extent of the author’s knowledge, no randomized clinical trial has compared the efficacy of ketorolac alone versus its combination with tramadol/acetaminophen administered orally to control pain intensity after third molar surgery, which was the aim of the present investigation.

## Material and Methods

This randomized controlled clinical trial was approved by the ethics committee of State University of Ponta Grossa, Ponta Grossa, Paraná, Brazil (≠ 1.449.613), registered in the Brazilian Registry of Clinical Trials (≠ RBR-3phy2q) and prepared using the protocol established by the Consolidated Standards of Reporting Trials Statement (14).

All participants included in this study signed a free and informed consent form and underwent surgery during sessions that were part of the Clinic of Oral and Maxillofacial Surgery in the Department of Oral and Maxillofacial Surgery and Dental Specialty Center of the State University of Ponta Grossa, Brazil. This study was performed from March 8th, 2016, to December 14th, 2016, in the city of Ponta Grossa (Paraná, Brazil).

Participants with classification of surgical risk ASA 1 (American Society of Anesthesiologists) were selected, with indication of treatment for bilateral third-molar removal, in similar inclusion pattern. Orthopantomograms were taken to ensure similarity of the tooth inclinations. Tooth inclinations were determined using the classifications provided by Winter, using only the vertical and mesioangular positions. We included position “B” and class “II” relationship, which was based on the Pell and Gregory classification; with an extraction degree of difficulty from mild to moderate and at least 1/3 of the root formed, according to the radiographic evaluation, ensuring a lower variability in surgical trauma during the extraction on both sides (right and left) in each patient selected.

Patients with a history of hypersensitivity to medications used in the study; pregnant women, asthmatics, diabetics, infants, hypertensives, patients with gastrointestinal disorders (ulcer and bleeding), myasthenia gravis, glaucoma, patients who were immunosuppressed, dependent on narcotic drugs, who had neurological and/or behavioral changes, users of anti-inflammatories or antihypertensive drugs were not included in the study.

The study followed a crossover design, i.e., a single patient was submitted to two different pharmacological protocols for postoperative pain control.

The participants in Group K+T+A – received 1 tablet of Ketorolac 10 mg (Toragesic®, EMS Farmacêutica, Hortolândia, São Paulo, Brazil) plus 1 capsule of Tramadol 37.5 mg/acetaminophen 325 mg (Amanda Pharmaceutical Manipulations®, Ponta Grossa, Paraná, Brazil) under oral route 1 h before surgery and every 6 h for 48 h. In Group K – received 1 tablet of Ketorolac 10 mg (Toragesic®) plus 1 placebo capsule (Amanda Pharmaceutical Manipulations®), under oral route 1 h before surgery and every 6 h for 48 h.

Sample size calculation was done by the website www.sealedenvelope.com, using the primary outcome pain intensity of postoperative pain. To determine if oral ketorolac is as effective as its association with tramadol/acetaminophen (considering an equivalence limit of 20 units of the 0-100 visual analog scale (VAS); 90% power and type I error of 5%), this equivalence clinical trial required a minimum sample size of 46 participants, but 52 healthy individuals were selected for this clinical trial.

The randomization of the groups was performed through the generation of a list that determined the group of the first surgery. This random order of surgeries was kept in opaque and sealed envelopes, which were numbered sequentially. Immediately before the start of the first surgery, the side to be operated was determined by the coin toss. The envelope was opened to reveal the group, so the other side to be operated would receive treatment from the other group, one month after, respecting the washout period between treatments. To keep the operator, evaluator and patient blind, all medicines, were placed in identical capsules, and encoded by an independent investigator, not involved in the surgical and assessment steps. The surgeon and participant were not aware, at any time, of which drug was administered to the chosen side. Antibiotic prophylaxis with pre-administration of amoxicillin 1 g (Generic, Tetuo ® - Anápolis, GO, Brazil) or clindamycin 600 mg (Generic, Tetuo ® - Anápolis, GO, Brazil), in those participants allergic to amoxicillin, was given orally 1 h prior to surgery.

The surgical procedure was performed according to the principles of third molar removal surgery, from asepsis to synthesis, using routine materials and instruments required for this surgical practice. The same surgical technique was performed on both sides. Anesthesia was performed through the regional block of the inferior alveolar, lingual and buccal nerve using the same anesthesia (mepivacaine 2% with epinephrine 1: 100.000 - Mepiadre - Nova DFL®, Rio de Janeiro, RJ, Brazil).

Two types of rescue medication were prescribed to be used if needed.

1) tablets of acetaminophen 500 mg (Generic, Tetuo ® - Anápolis, GO, Brazil), with instructions to take 1 tablet of the drug in case of pain every 6 h; and 

2) tablets of ondansetron 4 mg (Vonau flash, Biolab®, Taboão da Serra, SP, Brazil), with instructions to take 1 tablet of the drug in case of nausea. The participants were also asked to take notes (day and time) whenever consuming these drugs in an appropriate questionnaire form given by the surgeon after each surgery.

-Pain assessments

Analgesic efficacy was assessed based on four key endpoints, which the patients were required to record on a pain diary:

Pain intensity 

Participants were instructed to record their postoperative pain intensity (immediately, 3, 6, 9, 12, 24 and 48 h after surgery) on a VAS 0 to 100 mm scale, where 0 = no pain and 100 = unbearable pain.

Total number of rescue medication consumption 

Participants were also instructed to record the total amount of analgesics (acetaminophen 500 mg tablets) or antiemetic (ondansetron 4 mg) consumed during the evaluation period (48 h) in their form.

Global assessment

Participants were asked to provide an overall evaluation of the efficacy of the surgery regarding pain on a five-point categorical scale at the end of the trial. The categories of scale were 1 = poor, 2 = fair, 3 = good, 4 = very good and 5 = excellent, in which excellent means minimum pain and poor means severe pain (15).

Assessment of adverse effects 

Participants were asked to include the occurrence of some common side effects such as dizziness, nausea, vomiting, stomachaches or other gastrointestinal discomforts, migraines or effects. The participants were also instructed to note any rarer occurrences such as prolonged bleeding after surgery, renal problems and/or other gastrointestinal disorders (9).

-Statistical analysis

The need for rescue medication (paracetamol and ondansetron) was analyzed by the McNemar’s test. The intensity of pain in each time period, the consumption of rescue medication and the evaluation of the overall effect for the two groups were performed with the Wilcoxon Signed-Rank test. Within each group, pain intensity at different periods was compared with the Friedman and Student Neuman’s tests. All tests were at a significance level of 0.05 with the software Sigma Plot Software program (Systat Software, San Jose, California, USA).

## Results

Three out of the 52 patients did not perform the second surgery due to postoperative complications in the first surgery. The patients were immediately treated, and the complications were controlled, but they did not want to do the second surgery. Two complications were related to the surgical procedure: paresthesia (Group K) and infection (Group K+T+A). The other complication (Group K+T+A) occurred due to gastrointestinal symptoms (the patient had nausea and vomiting, she went to a hospital and, during treatment, she had an extrapyramidal reaction due to metoclopramide). All patients recovered well from these adverse effects.

Baseline characteristics

**Figure 1:**
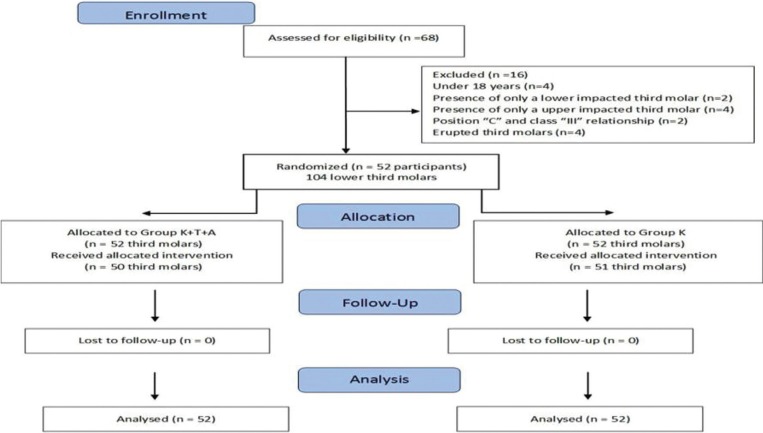
Flow diagram of the clinical trials including detailed information on the excluded participants.

A total of 52 patients were selected (Fig. 1). The overall mean age was 20.8 ± 3.2 years (ranging from 18 and 35 years), 77% were women, 5.7% were smokers, the overall mean weight was 59.8 ± 9.8, the overall mean height was 166.8 ± 7.9 and 100% were white. Significant differences were observed among the different time assessments (*p* < 0.05;  Table 1). The level of pain increased after surgery, reaching a peak after 3 h for both groups. In the following hours, pain intensity started to decrease, but few patients reported pain 48 h after the surgery ( Table 1). The comparison of both groups in each time assessment only showed significant differences in the 9-h period, with a lower level of pain intensity for group K+T+A than group K.

**Table 1:**
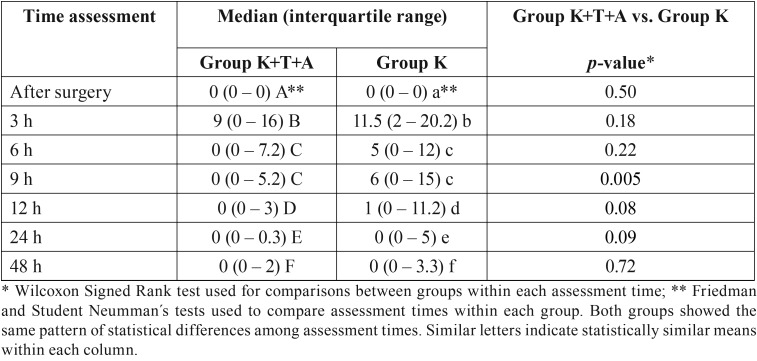
Pain intensity (median and interquartile range) of the group at the different time assessments along with the statistical comparisons.

The mean difference of pain intensity in the different assessment periods varied from approximately 4 to 7 units in a 0 to 100 VAS scale ( Table 2).

**Table 2:**
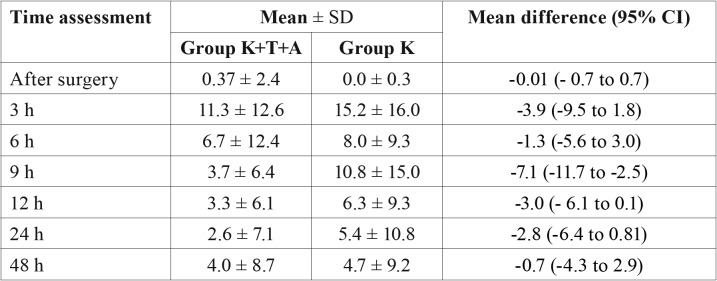
Pain intensity (mean ± standard deviations [SD]) of the group at the different time assessments along with effect size.

The need for analgesics in Group K was significantly greater than that for Group K+T+A (*p* < 0.001). However, the need for antiemetics in Group K+T+A was greater than for Group K (*p* < 0.0001). No differences in the median number of pills for both rescue medicines (analgesics and antiemetics) were observed between groups (*p* > 0.06) ( Table 3).

**Table 3:**
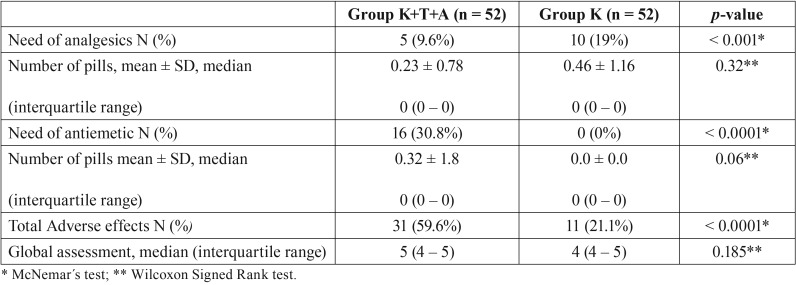
Comparison of the need of rescue analgesics, antiemetics, total number of adverse effects and global assessment.

The patient’s overall assessment of the surgery in relation to pain is shown in  Table 3. No significant differences between groups were observed, and most of the patients reported that the overall assessment was very good and excellent.

With the exception of three participants, as mentioned earlier. No other patient had serious adverse events in any of the study groups. However, the total number of adverse effects was higher in Group K+T+A ( Table 3). The most common types of adverse effects are seen in  Table 4. From all the adverse effects described, dizziness (*p* = 0.002), nausea (*p* = 0.0001) and vomiting (*p* = 0.0009) were statistically more common in Group K+T+A than in Group K ( Table 4).

**Table 4:**
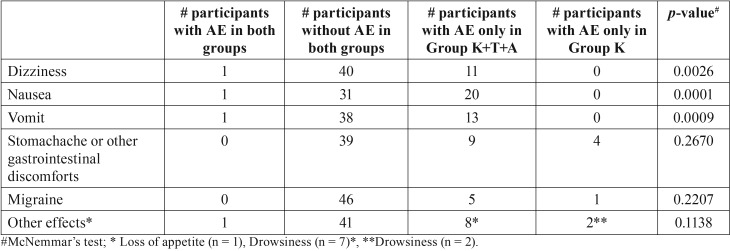
Comparison of the number of participants with adverse effects (AE).

## Discussion

Differently than other studies that administered only one dose of the medication before (15,16) or after (11) surgery, we employed a perioperative protocol in this study. A single-dose regimen may provide the fall of drug levels in the blood plasma, while a multiple-dosage regimen may achieve relatively constant plasma concentrations of the drug within the limits of its therapeutic window. Considering that the duration of analgesia of tramadol/acetaminophen (13) and ketorolac (17) was reported to be 5 h and 6 h, respectively, the patients were instructed to take the medications every 6 h for 48 h.

The strategy of pre-surgical analgesic administration, as performed in this study (1 h before surgery), was used in theory to establish effective blood levels for maximum analgesic effect at the time pain is most severe.

Postoperative pain intensity after third molar surgery is moderate to severe, usually starts within the first 3 h and reaches peaks after approximately 6 to 8 h. The highest pain levels are recorded within the first 12 h (15,18). From this period on, pain intensity starts to reduce, reaching very low levels after 48 h. It is reported that this peak of pain, during the early postoperative period, coincides with the increased production of biochemical mediators of pain at the surgical site (19).

The worst scenario of pain was detected at the 3-h period in the present investigation, with an average pain intensity of 15 units in the VAS scale in group K. The higher intensity at 3 h compared to the other time assessments may be due to the fact that we used a medium-duration anesthetic with a 2.5-h duration of analgesia (20). Therefore, this period of maximum pain intensity coincides with the conclusion of the period of painlessness as normal sensations return and when there is an increased release of pain mediators (21).

The purpose of using a combination of analgesics with different mechanisms of action is to achieve a synergistic potentiation (22), yielding a sufficient analgesic effect with lower doses and therefore reducing the intensity and incidence of side effects (23). In this study, at the 9-h period, a significant difference was found between the groups, with lower pain intensity for the group that associated ketorolac with analgesics. The synergism that occurs with both drugs that act on different mechanisms of pain perception may adequately explain this pain reduction.

The major mechanism by which ketorolac exerts its pharmacological effects is predominantly through the peripheral inhibition of prostaglandin synthesis through cyclooxygenase-1 and -2 inhibition (24). Ketorolac is most active in the periphery and only shows mild central nervous system effects at doses far greater than those required for analgesic and antiinflammatory activity (25).

Tramadol hydrochloride, which has a weak opioid activity, produces analgesia through an opioid effect that binds (μ)-opioid receptors and modifies the transmission of pain signals through the inhibition of serotonin and norepinephrine reuptake within pain pathways of the central nervous system (13).

Acetaminophen, on the other hand, produces analgesia by elevating the pain threshold through inhibition of N-methyl D-aspartate or substance P-mediated nitric oxide synthesis and/or inhibition of prostaglandin E2 release in the central nervous system (13).

The mechanisms responsible for the synergism in the analgesic activity of acetaminophen/NSAIDs combinations are not clear. But it seems to involve several mechanisms that are probably implicated in the antinociceptive activities, many of them at central levels producing a supra-additive or synergic analgesic effect (26). In animal models of neuropathic and inflammatory pain, there is some evidence for synergistic potentiation between opioids and NSAIDS (27).

Although lower pain at 9 h was observed for group K+T+A, this synergism should be viewed with caution because, from a clinical perspective, this difference (or effect size) was of a small magnitude and its benefits did not overcome the greater number of side effects in the combination drug group.

Adverse effects of oral ketorolac were mild in intensity and well tolerated, as well as reported by other authors (15,28). Stomachaches or other gastrointestinal discomforts, somnolence and migraines were observed in the ketorolac group, as previously reported (28). The most common adverse effects of tramadol/acetaminophen in lower dosages (like the one used in the present investigation) were nausea, dizziness, vomiting, stomach aches or other gastrointestinal discomforts, loss of appetite and somnolence. There were no serious adverse events reported for any of the study groups.

An antiemetic drug (Ondansetron 4 mg) was also used as a rescue medication for the control of nausea in patients. In group K+T+A, 30.8% of the patients took the antiemetic, in contrast with only 0% from group K. The higher percentage in the association group may be justified by the fact that tramadol induces nausea and vomiting by stimulation of the chemoreceptor trigger zone (CTZ) richly endowed with serotonin receptors. Serotonin stimulates the vomiting center and transmits signals through the stomach, small intestine, diaphragm and abdominal musculature, thus increasing the intragastric pressure that provokes nausea and vomiting.

The need of rescue medication for analgesia (acetaminophen) in the ketorolac group (19%) was significantly greater than that observed in the association group (9.6%). The associated group required less rescue medication, probably because of more analgesic potency from the basic medication.

Various factors such as: the preemptive dose, the multiple-dose regimens, the same expert surgeon and surgical technique for all procedures, the same surgical difficulty in terms of the magnitude of surgical trauma on both sides of the mandible and the similar amount of anesthetic volume used in anesthesia techniques might in part explain the low amount of analgesics used postoperatively, compared to single dose regimes (9,15).

The groups presented similar results regarding the overall assessment, which can be explained by the overall low intensity of pain in both groups. In the worst pain situation, where the pain peak occurs, pain intensity ranged from approximately 4-10% of the maximum VAS pain.

We cannot rule out the fact that the use of rescue medication adds an additional variable to the research design and may lead to overestimation of the beneficial effect of the group that took more rescue medication. However, with the growing rigor of research ethics committees, it is not currently possible to carry out any research that may submit the participants to painful or other types of suffering that could be avoided. This also explains why previous clinical trials on this issue also employed rescue medication (29). Thus, rescue medication is imperative in studies that test analgesic control. This was compensated for by comparing the amount of rescue medication used in both group.

## Conclusions

Both ketorolac alone and ketorolac plus tramadol/acetaminophen showed good control of pain after extraction of the lower third molars. Although the VAS score in the association group was statistically lower at 9 h, the pain difference is small and not clinically relevant, and the association is more expensive and caused more side effects.

## References

[1] Haraji A, Rakhshan V (2015). Chlorhexidine gel and less difficult surgeries might reduce post-operative pain, controlling for dry socket, infection and analgesic consumption: a split-mouth controlled randomised clinical trial. J Oral Rehabil.

[2] Osunde OD, Adebola RA, Omeje UK (2011). Management of inflammatory complications in third molar surgery: a review of the literature. Afr Health Sci.

[3] López Carriches C, Martínez González JM, Donado Rodríguez M (2006). The use of methylprednisolone versus diclofenac in the treatment of inflammation and trismus after surgical removal of lower third molars. Med Oral Patol Oral Cir Bucal.

[4] Ong CK, Seymour RA (2003). Pathogenesis of postoperative oral surgical pain. Anesth Prog.

[5] Vegas-Bustamante E, Mico-Llorens J, Gargallo-Albiol J, Satorres-Nieto M, Berini-Aytes L, Gay-Escoda C (2008). Efficacy of methylprednisolone injected into the masseter muscle following the surgical extraction of impacted lower third molars. Int J Oral Maxillofac Surg.

[6] Forbes JA, Kehm CJ, Grodin CD, Beaver WT (1990). Evaluation of ketorolac, ibuprofen, acetaminophen, and an acetaminophen-codeine combination in postoperative oral surgery pain. Pharmacotherapy.

[7] McAleer SD, Majid O, Venables E, Polack T, Sheikh MS (2007). Pharmacokinetics and safety of ketorolac following single intranasal and intramuscular administration in healthy volunteers. J Clin Pharmacol.

[8] Flores-Murrieta FJ, Granados-Soto V (1996). Pharmacologic Properties of Ketorolac Tromethamine: A Potent Analgesic Drug. CNS Drug Reviews.

[9] Mehlisch DR, Desjardins PJ, Daniels S, Hubbard RC (2003). Single doses of parecoxib sodium intravenously are as effective as ketorolac in reducing pain after oral surgery. J Oral Maxillofac Surg.

[10] Trindade PA, Giglio FP, Colombini-Ishikiriama BL, A Calvo AM, Modena KC, Ribeiro DA (2012). Sublingual ketorolac and sublingual piroxicam are equally effective for postoperative pain, trismus, and swelling management in lower third molar removal. Oral Surg Oral Med Oral Pathol Oral Radiol.

[11] Raffa RB (2001). Pharmacology of oral combination analgesics: rational therapy for pain. J Clin Pharm Ther.

[12] Au AH, Choi SW, Cheung CW, Leung YY (2005). The Efficacy and Clinical Safety of Various Analgesic Combinations for Post-Operative Pain after Third Molar Surgery: A Systematic Review and Meta-Analysis. PLoS One.

[13] Jung YS, Kim DK, Kim MK, Kim HJ, Cha IH, Lee EW (2004). Onset of analgesia and analgesic efficacy of tramadol/acetaminophen and codeine/acetaminophen/ibuprofen in acute postoperative pain: a single-center, single-dose, randomized, active-controlled, parallel-group study in a dental surgery pain model. Clin Ther.

[14] Schulz KF, Altman DG, Moher D, CONSORT Group (2011). CONSORT 2010 statement: updated guidelines for reporting parallel group randomised trials. Int J Surg.

[15] Ong KS, Tan JM (2004). Preoperative intravenous tramadol versus ketorolac for preventing postoperative pain after third molar surgery. Int J Oral Maxillofac Surg.

[16] Isiordia-Espinoza MA, Pozos-Guillen A, Martinez-Rider R, Perez-Urizar J (2016). Comparison of the analgesic efficacy of oral ketorolac versus intramuscular tramadol after third molar surgery: A parallel, double-blind, randomized, placebo-controlled clinical trial. Med Oral Patol Oral Cir Bucal.

[17] Ong KS, Seymour RA, Chen FG, Ho VC (2004). Preoperative ketorolac has a preemptive effect for postoperative third molar surgical pain. Int J Oral Maxillofac Surg.

[18] Ong CK, Seymour RA (2008). An evidence-based update of the use of analgesics in dentistry. Periodontol 2000.

[19] Paiva-Oliveira JG, Bastos PR, Cury Pontes ER, da Silva JC, Delgado JA, Oshiro-Filho NT (2016). Comparison of the anti-inflammatory effect of dexamethasone and ketorolac in the extractions of third molars. Oral Maxillofac Surg.

[20] Colombini BL, Modena KC, Calvo AM, Sakai VT, Giglio FP, Dionisio TJ (2006). Articaine and mepivacaine efficacy in postoperative analgesia for lower third molar removal: a double-blind, randomized, crossover study. Oral Surg Oral Med Oral Pathol Oral Radiol Endod.

[21] Tuzuner Oncul AM, Yazicioglu D, Alanoglu Z, Demiralp S, Ozturk A, Ucok C (2011). Postoperative analgesia in impacted third molar surgery: the role of preoperative diclofenac sodium, paracetamol and lornoxicam. Med Princ Pract.

[22] Tallarida RJ (2001). Drug synergism: its detection and applications. J Pharmacol Exp Ther.

[23] Ortiz MI, Molina MA, Arai YC, Romano CL (2012). Analgesic drugs combinations in the treatment of different types of pain. Pain Res Treat.

[24] Galan-Herrera JF, Poo JL, Maya-Barrios JA, de Lago A, Oliva I, Gonzalez-de la Parra M (2008). Bioavailability of two sublingual formulations of ketorolac tromethamine 30 mg: a randomized, open-label, single-dose, two-period crossover comparison in healthy Mexican adult volunteers. Clin Ther.

[25] Rooks WH 2nd (1990). The pharmacologic activity of ketorolac tromethamine. Pharmacotherapy.

[26] Miranda HF, Puig MM, Prieto JC, Pinardi G (2006). Synergism between paracetamol and nonsteroidal anti-inflammatory drugs in experimental acute pain. Pain.

[27] Lopez-Munoz FJ, Diaz-Reval MI, Terron JA, Deciga-Campos M (2004). Analysis of the analgesic interactions between ketorolac and tramadol during arthritic nociception in rat. Eur J Pharmacol.

[28] Mishra H, Khan FA (2012). A double-blind, placebo-controlled randomized comparison of pre and postoperative administration of ketorolac and tramadol for dental extraction pain. J Anaesthesiol Clin Pharmacol.

[29] da Costa Araujo FA, de Santana Santos T, de Morais HH, Laureano Filho JR, de Oliveira ESED, Vasconcellos RJ (2012). Comparative analysis of preemptive analgesic effect of tramadol chlorhydrate and nimesulide following third molar surgery. J Craniomaxillofac Surg.

